# Supplementary material for: Comparison of Immune-Related Gene Expression in Two Chicken Breeds Following Infectious Bronchitis Virus Vaccination
Source: Animals (Basel). 2023 May 15;13(10):1642. doi: 10.3390/ani13101642 (PMC10215283; doi:10.3390/ani13101642)
Supplement: Supplementary file 1 [file animals-13-01642-s001.zip › animals-2287908-supplementary.pdf]

Table S1. Primer sequences used for quantitative real-time polymerase chain reaction analysis.

Gene	Forward primer	Reverse primer	Accession number
IL-6	AAATCCCTCCTCGC CAATCT	CCCTCACGGTCTTCTCCATAAA	ENSGALG00000010915
IL-22	TGTTGTTGCTGTTTC CCTCTTC	CACCCCTGTCCCTTTTGGA	ENSGALG00000009904
CXCL13L2	GCCTTTCCCCAGCT CCATAG	AGACCTCAGTGTCTCGTGCT	ENSGALG00000010338
CXCR 4	GGCTGCCGTATTAC ATTGGC	GCAATGGAAGAACGCAAGGG	ENSGALG00000012357
CCL19	GAAACGCCTGGAA CTCTTGC	AGCCTAGAGCAGGGATTGGA	ENSGALG00000028256
CD34	GGGAGAGTTGTTCC GTCTGT	GAGTTCATGGCAACCTCGCT	ENSGALG00000001177
GAPDH	ACTGTCAAGGCTGA GAACGG	CATTTGATGTTGCTGGGGTC	NM_204305

Table S2. Sequencing data of Taiwan Country chicken and White Leghorn chicken of day 7 postvaccination.

Sample	Clean Read	Clean base	Q30 (%)	GC content (%)
**BC31**	36735506	5.31 G	93.915	46.11
**BC32**	36243634	5.20 G	93.875	45.615
**BC33**	42910656	6.18 G	93.585	46.645
**BT31**	42386752	6.13 G	94.07	46.305
**BT32**	40173510	5.81 G	93.64	46.685
**BT33**	35954314	5.21 G	93.865	45.61
**WC31**	35972344	5.20 G	93.97	46.025
**WC32**	35980810	5.21 G	93.565	45.91
**WC33**	37254966	5.39 G	93.705	46.545
**WT31**	39261272	5.68 G	93.6	46.255
**WT32**	36290734	5.25 G	93.87	44.79
**WT33**	51867002	7.55 G	94.395	45.025

Note: Clean reads: total number of pair-end reads in the clean data; Clean bases: total number of bases in the clean data; GC content: percentage of G and C bases in the clean data; % Q30: the percentage of Q30 base.

Table S3. Comparison of results from Taiwan Country chicken and White Leghorn chicken of day 7 postvaccination.

Sample	Total reads	Unmapped	Total mapped	Multiple mapped	Uniquely mapped
**BC31**	36735506 (100 %)	1823128 (4.96 %)	34912378 (95.04 %)	1715786 (4.67 %)	33196592 (90.37 %)
**BC32**	36243634 (100 %)	2461672 (6.79 %)	33781962 (93.21 %)	1031568 (2.85 %)	32750394 (90.36 %)
**BC33**	42910656 (100 %)	2325747 (5.42 %)	40584909 (94.58 %)	2898449 (6.75 %)	37686460 (87.83 %)
**BT31**	42386752 (100 %)	2311461 (5.45 %)	40075291 (94.55 %)	2040456 (4.81 %)	38034835 (89.73 %)
**BT32**	40173510 (100 %)	2057588 (5.12 %)	38115922 (94.88 %)	2017934 (5.02 %)	36097988 (89.86 %)
**BT33**	35954314 (100 %)	1819678 (5.06 %)	34134636 (94.94 %)	1146963 (3.19 %)	32987673 (91.75 %)
**WC31**	35972344 (100 %)	1654467 (4.6 %)	34317877 (95.4 %)	1716067 (4.77 %)	32601810 (90.63 %)
**WC32**	35980810 (100 %)	1663271 (4.62 %)	34317539 (95.38 %)	1896180 (5.27 %)	32421359 (90.11 %)
**WC33**	37254966 (100 %)	1802720 (4.84 %)	35452246 (95.16 %)	2415928 (6.48 %)	33036318 (88.68 %)
**WT31**	39261272 (100 %)	1715663 (4.37 %)	37545609 (95.63 %)	2217391 (5.65 %)	35328218 (89.98 %)
**WT32**	36290734 (100 %)	1841341 (5.07 %)	34449393 (94.93 %)	796201 (2.19 %)	33653192 (92.73 %)
**WT33**	51867002 (100 %)	2485172 (4.79 %)	49381830 (95.21 %)	2075873 (4 %)	47305957 (91.21 %)

Note: Total Reads: the number of single-end reads in the clean data; Unmapped: the number of reads that cannot find the match on the reference genome and the percentage of unmapped reads in the clean reads; Total Mapped: the number of reads on the reference genome and the percentage of mapped reads in the clean reads; Multiple Mapped: the number of reads compared with the multiple locations of the reference genome and the percentage of multiple map reads in the clean reads; Uniquely Mapped: the number of reads compared with the only location of the reference genome and the percentage of clean reads.

Table S4. Gene ontology analysis of differentially expressed genes between vaccinated Taiwan Country chicken and unvaccinated Taiwan Country chicken (TCC^{Vac} VS TCC^{Unvac}) at day 7 postvaccination.

GO accession	Description	p-value	Count	Genes ID
GO:0042221	Response to chemical	7.49×10^{-5}	24	HSPA5/PRLR/LECT2/HSPA8/CEBPB/ FLRT3/ IL6/HSPA2/ESR2/PDGFRA/ACTN2/GHR / CHRNA6/AvBD2/AvBD1/HBBA/SPIK5/ HBM/NFASC/HSP90AA1/AGTR1/ RGMA/ MSTN/HBA1
GO:0009725	Response to hormone	1.35×10^{-4}	10	PRLR/IL6/HSPA2/ESR2/ACTN2/GHR/SPIK5/ HSP90AA1/AGTR1/MSTN
GO:0042330	Taxis	9.37×10^{-3}	6	FLRT3/PDGFRA/AvBD2/AvBD1/NFASC /MSTN
GO:1901653	Cellular response to peptide	1.19×10^{-2}	4	ACTN2/GHR/AGTR1/MSTN

^{Vac}Vaccinated group; ^{Unvac}Unvaccinated group.

Table S5. Kyoto Encyclopedia Analysis of Genes and Genome pathway analysis of differentially expressed genes between vaccinated Taiwan Country chicken and unvaccinated Taiwan Country chicken (TCC^{Vac} VS TCC^{Unvac}) at day 7 postvaccination.

ID	Description	p-value	Count	Symbol
gga04060	Cytokine-cytokine receptor interaction	$4.49×10^{-6}$	15	PRLR/CXCL14/IL18/IL22/CXCL13L2/CXCL13L3/IL6/LEPR/GHR/IFNKL1/CCL19/CCL4/MSTN/CCL5/CXCL13
gga04623	Cytosolic DNA-sensing pathway	$1.36×10^{-3}$	5	IL18/IL6/IFNKL1/CCL4/CCL5
gga05164	Influenza A	$4.47×10^{-3}$	8	PLG/IL18/IL6/IFNKL1/MX1/RSAD2/CCL5/PMLL
gga04621	NOD-like receptor signaling pathway	$9.72×10^{-3}$	7	IL18/IL6/MAPK10/IFNKL1/AvBD2/HSP90AA1/CCL5
gga04620	Toll-like receptor signaling pathway	$2.13×10^{-2}$	5	IL6/MAPK10/IFNKL1/CCL4/CCL5
gga04080	Neuroactive ligand-receptor interaction	$2.31×10^{-2}$	12	GABRA1/TSPO2/PRLR/PLG/NPY4R/LEPR/GHR/F2R/ CHRNA6/ADRA2A/AGTR1/GRP
gga04920	Adipocytokine signaling pathway	$3.29×10^{-2}$	4	CPT1A/LEPR/MAPK10/PPARGC1A

^{Vac}Vaccinated group; ^{Unvac}Unvaccinated group.

Table S6. Gene ontology analysis of differentially expressed genes between vaccinated White Leghorn chicken and unvaccinated White Leghorn chicken (WLC^{Vac} VS WLC^{Unvac}) at day 7 postvaccination.

GO accession	Description	p-value	Count	Genes ID
GO:0032675	Regulation of interleukin-6 production	2.70×10^{-2}	1	IL6
GO:0032635	Interleukin-6 production	3.00×10^{-2}	1	IL6
GO:0042102	Positive regulation of T cell proliferation	3.00×10^{-2}	1	IL6
GO:0050871	Positive regulation of B cell activation	3.00×10^{-2}	1	IL6
GO:0050864	Regulation of B cell activation	3.50×10^{-2}	1	IL6

^{Vac}Vaccinated group; ^{Unvac}Unvaccinated group.

Table S7. Kyoto Encyclopedia Analysis of Genes and Genome pathway analysis of differentially expressed genes between vaccinated White Leghorn chicken and unvaccinated White Leghorn chicken (WLC^{Vac} VS WLC^{Unvac}) at day 7 postvaccination.

ID	Description	p-value	Count	Symbol
gga04080	Neuroactive ligand-receptor interaction	3.00×10^{-3}	6	UTS2R/TSPO2/OPRL1/MC5R/GRM8/NPW
gga00430	Taurine and hypotaurine metabolism	4.60×10^{-2}	1	GADL1
gga00360	Phenylalanine metabolism	6.30×10^{-2}	1	IL4I1
gga04060	Cytokine-cytokine receptor interaction	6.50×10^{-2}	3	IL6/BMP6/AMH
gga04350	TGF-beta signaling pathway	6.50×10^{-2}	2	BMP6/AMH
gga04145	Phagosome	1.30×10^{-1}	2	ATP6V0D2/EPX

^{Vac}Vaccinated group; ^{Unvac}Unvaccinated group.
